# The influence of self-efficacy and satisfaction with e-learning on academic performance among nursing students: a multisite study

**DOI:** 10.1186/s12909-026-09236-1

**Published:** 2026-04-24

**Authors:** Adnan Innab, Essa Hakamy, Nedaa Abdulgafor, Nora Ghalib AlOtaibi

**Affiliations:** 1 Nursing Administration and Education Department, College of Nursing, King Saud University, Riyadh, 12372 Saudi Arabia; 2 Nursing Administration and Education Department, King Saud University, Riyadh, 12372 Saudi Arabia; 3 Department of Community Nursing, College of Nursing, Imam Abdulrahman Bin Faisal University, Dammam, Saudi Arabia; 4 Nursing College, King Saud University, Riyadh, 12372 Saudi Arabia; 5 Nursing Administration and Education Department, King Saud University, P.O. Box 642, Riyadh, 11421 Saudi Arabia

**Keywords:** e-learning satisfaction, Self-efficacy, Academic performance, GPA

## Abstract

**Background:**

Academic performance and self-efficacy are closely related within educational systems. Students with higher self-efficacy across various subject areas typically exhibit higher academic achievement and students who adopt a variety of learning strategies are more likely to demonstrate high-order learning behaviors and skills. However, evidence on the relationships among e-learning satisfaction, self-efficacy, and academic performance remains inconclusive. This study was guided by Albert Bandura’s theory of self-efficacy.

**Aim:**

This study aimed to assess self-efficacy, satisfaction with e-learning, and perceived academic performance among nursing students engaged in synchronous virtual learning.

**Methods:**

This was a multisite cross-sectional, correlational, study. The authors adhered to the Strengthening the Reporting of Observational Studies in Epidemiology (STROBE) checklist.

A convenience sampling method was used to recruit the participants from different universities nationwide. Data were collected from 163 nursing students using the student outcome survey, General Self-Efficacy Scale (GSE), and perceived academic performance scale. Data were analyzed using independent sample t-test, Pearson’s product moment correlation, and multiple linear regression.

**Results:**

The overall e-learning satisfaction was relatively low. Participants generally reported moderate to high levels of general self-efficacy. Undergraduate students had greater satisfaction with e-learning (M= 3.06, SD = .69, *p*<.01) and higher perceived academic performance (M= 3.46 , SD= .67, *p* <.05) compared to graduate students. General self-efficacy was significantly associated with satisfaction with e-learning (r = .579, *p* < .001) and academic performance (r = .511, *p* < .001). The regression analysis explained 35% of the variance and indicated that both general self-efficacy (β = 0.047, *p*<.001) and satisfaction with e-learning (β = .37, *p* <.001) were significantly associated with perceived academic performance.

**Conclusions:**

The present results underscore the critical roles of self-efficacy and e-learning satisfaction in relation to academic performance, which is consistent with Bandura’s self-efficacy theory. These insights can inform targeted interventions to strengthen these factors, ultimately improving nursing students’ academic performance and overall e-learning satisfaction

## Background

In recent years, higher education has shifted from traditional teacher-centered approaches to more dynamic, student-centered opportunities, including e-learning [1]. Although many institutions have previously adopted this approach [2], the COVID-19 pandemic accelerated the transition in numerous countries, including Saudi Arabia, to ensure students’ safety and well-being [3, 4]. Subsequently, the Saudi Arabian Ministry of Education promoted a rapid shift to e-learning environments [5]. E-learning utilizes electronic media, such as computers, the internet, multimedia CDs, and electronic journals, to reduce demands on traffic, physical space, time, and finances while making learning faster and more accessible [6].

Self-regulated learners are essential in contemporary educational contexts amid rapid systemic changes, and self-efficacy plays a crucial role in these contexts [7]. As Bandura’s [31] theory posited, educational settings are highly conducive to developing self-efficacy. Self-efficacy refers to an individual’s evaluation of their ability to successfully perform a specific task. In achievement-oriented academic settings, it denotes a student’s confidence in accomplishing a particular goal [8]. Self-efficacy fundamentally influences students’ decision-making, emotional investment, and persistence in the task [9]. Furthermore, it serves as a key factor in nursing students’ ability to engage with learning tasks, especially through e-learning platforms [10].

The primary objective at all educational levels is for students to succeed academically and achieve high grades, benefiting both students and educational institutions [11]. Academic performance measures students’ achievement across subjects, typically through objective measures like grade point average (GPA), test scores, or individual course grades [12]. Perceived academic performance, by contrast, represents students’ cognitive evaluation of their academic performance, encompassing grades, attitudes, and pathways to success within the learning environment [13, 14]. This perception stems from students’ subjective experiences, which are not captured by actual GPA, including factors such as self-efficacy, goal attainment, and contributions to well-being and educational processes. Academic performance provides deeper insight into students’ experiences [15].

Academic performance and self-efficacy in the education system are closely related within educational systems [16]. Previous researchers [17] studied 208 nursing students in Egypt using GPA, the Online Learning Readiness Scale, and the Academic Self-Efficacy Scale. Their findings revealed a substantial positive association between academic achievement and academic self-efficacy. Moreover, academic self-efficacy and e-learning readiness emerged as key predictors, accounting for 36% of the variance in academic achievement.

Prior research indicates that students with higher self-efficacy across various subject areas typically exhibit higher academic achievement [18] and students who adopt a variety of learning strategies are more likely to demonstrate high-order learning behaviors and skills [19]. Therefore, when academic self-efficacy is enhanced, strategic learning is activated; and effort and persistence increase, ultimately contributing to improved academic performance [20]. Satisfaction with e-learning reflects learners’ subjective experiences of the quality of services offered by online platforms [21, 22]. Learning satisfaction is determined by whether intended learning outcomes meet students’ expectations. Currently, e-learning satisfaction has emerged as a key research focus, drawing increasing scholarly attention [23, 24]. A previous study surveyed 1,255 health science students from 11 countries and found higher e-learning satisfaction [25] among those from developed countries (M = 7.34) than from developing countries (M = 5.82). Previous researchers reported that more than two-thirds of 219 nursing students in India were satisfied with e-learning [26]. By contrast, a study in Peru revealed that 54.4% of nursing students considered e-learning inadequate, associating it with poor academic performance [27]. Such discrepancies between developed and developing countries warrant further investigation.

Although previous research has linked self-efficacy, e-learning readiness, satisfaction with online learning, and academic achievement, it has not yet provided an integrated understanding of how these factors relate to one another [16, 17]. Much of the literature has examined these constructs in pairs, such as readiness with academic self-efficacy or satisfaction with academic outcomes, rather than exploring how students’ confidence beliefs and satisfaction with the online learning environment jointly relate to academic performance [5, 16, 17]. This distinction is particularly important in nursing education, where learning occurs in demanding academic and practice-oriented settings that require not only technological adaptation but also confidence, persistence, and active engagement [10, 28].

In addition, prior studies have often relied on objective indicators such as grade point average or academic achievement [16], whereas perceived academic performance may better reflect students’ own appraisal of their academic functioning, including confidence, adjustment, and perceived success within the learning environment [12, 14]. Existing evidence also suggests that academic self-efficacy is associated with academic achievement, while satisfaction has been linked to students’ perceived performance in e-learning settings, supporting the need for a more integrated model that brings these constructs together [14, 29].

A further gap concerns the learning context itself. Synchronous e-learning differs from asynchronous formats because it requires students to engage in real time, respond immediately to instructors and peers, and sustain participation during live online sessions [28, 30]. Recent evidence suggests that synchronous e-learning has distinct educational effects in nursing education, yet its relationship with students’ general self-efficacy, satisfaction with e-learning, and perceived academic performance remains insufficiently clarified [28, 30]. Therefore, this study aimed to assess self-efficacy, satisfaction with e-learning, and perceived academic performance among nursing students engaged in synchronous virtual learning. The specific aims were to (1) determine whether general self-efficacy (GSE) and satisfaction with e-learning are significantly associated with participants’ perceived academic performance (2), examine whether GSE and satisfaction with e-learning significantly predict participants’ perceived academic performance, and (3) identify mean differences in participants’ satisfaction with e-learning, GSE, and perceived academic performance according to demographic characteristics.

### Theoretical framework

**Fig. 1:**
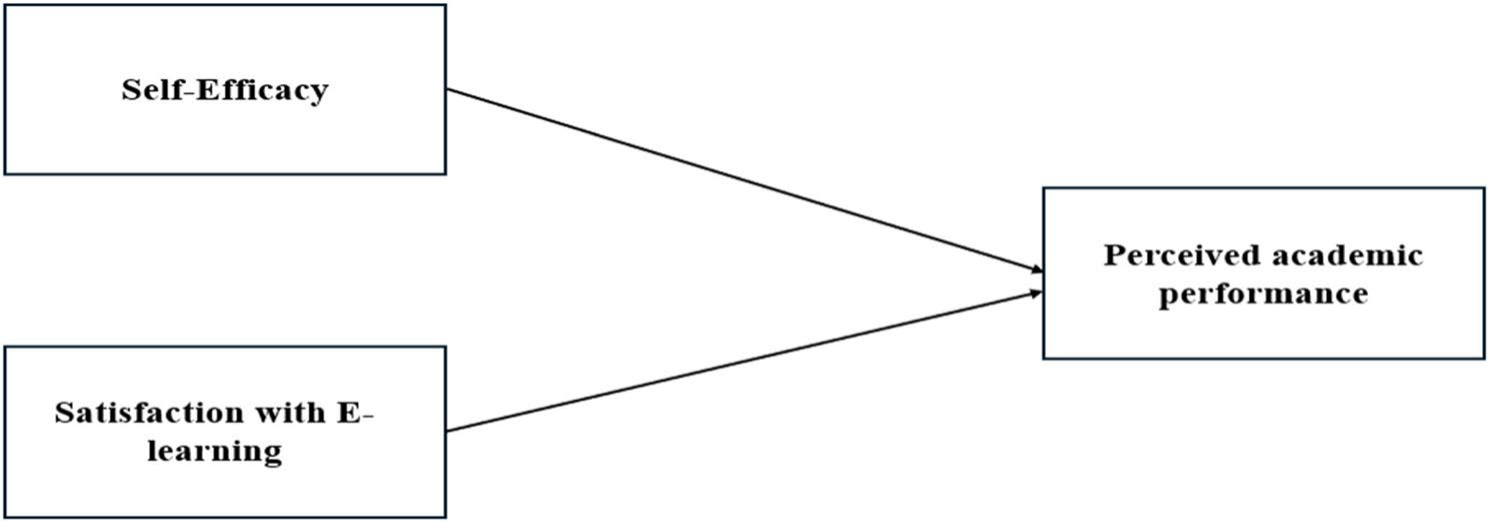
Hypothesized model of the influence of self-efficacy and satisfaction with e-learning on academic performance among nursing students

This study is grounded in Bandura’s self-efficacy theory, which posits that individuals’ beliefs in their capabilities are stronger predictors of behavior and outcomes than their actual abilities [31]. This theory supports the inclusion of self-efficacy as a key independent variable in the present study. Although Bandura’s work did not specifically address e-learning satisfaction, defined as students’ emotional responses to online learning environments [32], its emphasis on reciprocal interactions between personal and environmental factors provides a basis for its inclusion. In this study, general self-efficacy represents the personal factor, e-learning satisfaction reflects the contextual factor as students’ appraisal of the online learning environment, and perceived academic performance represents the outcome. Accordingly, the hypothesized model assumes that both self-efficacy beliefs and satisfaction with the e-learning context are relevant to understanding variation in perceived academic performance (Fig. 1).

## Methods

### Research design

This was a cross-sectional, correlational, descriptive study. The authors adhered to the Strengthening the Reporting of Observational Studies in Epidemiology (STROBE) checklist. Data were collected at a single point in time from multiple universities.

### Sample and settings

A sample of undergraduate and graduate students were invited to participate in the study. To ensure a diverse group of students from across the country, a convenience sampling method was used to recruit the participants. Students from different universities nationwide participated in the study.

G-Power 3.1.9 software (Heinrich-Heine-University, Düsseldorf, Germany) was used to determine the sample size. With a significance level of 0.05, a power of 0.90, an effect size of 0.15, and three variables, the minimum sample size required to conduct the regression analysis was determined to be 99. To avoid any issue with missing data, we decided to add 15% to the total sample size.

### Data collection procedure

The inclusion criteria were (1) an undergraduate or graduate nursing student enrolled in a course that uses synchronous learning in Saudi Arabia, and (2) proficient in English. Those who met the inclusion criteria and decided to participate in the study had full access to the online questionnaire.

A convenience sampling approach was used through online recruitment. An invitation was distributed via social media platforms, including Facebook, WhatsApp, Twitter, and student emails. The recruitment statement provided essential information about the study, including its purpose, the risks and benefits of participation, and the inclusion and exclusion criteria, along with the primary investigator’s contact information for human subjects concerns. However, recruitment through social media may have introduced self-selection bias by overrepresenting students who were more motivated or more digitally engaged. In addition, institutional variability was not controlled, which may further limit the representativeness of the sample and the generalizability of the findings to the broader target population.

### Measurements

Participants were asked questions regarding their age, gender, level of education, grade point average, and their previous attendance at training courses. The three instrument measurements were the Student Outcome Survey, General Self-Efficacy Scale, and Perceived Academic Performance Scale. The surveys were fully composed of previously validated instruments.

#### Student outcome survey

This self-report scale, initially developed to evaluate students’ satisfaction with their learning, encompasses 19 elements distributed across three subscales: teaching, assessment, general skills, and learning experiences [33]. All items on the scale were measured using a 5-point Likert scale ranging from 1 (strongly disagree) to 5 (strongly agree). Higher scores indicate greater satisfaction levels. In a previous study among nursing students in Saudi Arabia [34], the internal consistency for the subscales ranged from 0.77 to 0.82, demonstrating the robustness of the scale’s measurement properties. In this study, Cronbach’s alpha was 0.77, indicating that this tool is reliable.

#### General self-efficacy scale

The General Self-Efficacy Scale (GSE) is a psychological assessment tool designed to measure an individual’s belief in their ability to execute behaviors necessary to produce specific performance attainments [35]. It reflects a person’s confidence in handling various situations and their capacity to achieve goals. The GSE has been used in various contexts, including academic settings, clinical psychology, organizational behavior, and research studies focused on motivation and personal development [35, 36]. It comprises 10-items that are measured on a 4-point-Likert scale ranging from 1 = “not at all true” to 4 = “exactly true.” Higher scores indicate greater self-efficacy. The Cronbach’s alpha coefficient ranged from 0.76 to 0.90 [35], indicating acceptable internal consistency reliability.

#### Perceived academic performance

This scale was used to evaluate students’ perceptions of their academic performance [37]. It consists of 5 items that are measured on a 5-point Likert scale, ranging from 1 (do not agree at all) to 5 (strongly agree). Higher scores indicated a greater perception of their academic performance. This scale has been used in mental health research and among different disciplines (e.g., political sciences, communication, actuarial sciences, sociology; [37, 38]. The scale has shown acceptable internal consistency reliability (Cronbach’s α = 83).

### Ethical considerations

Approval from the Institutional Review Board at King Saud University was secured prior to data collection [*KSU-REC 006QS-E]*. The study adhered to the Declaration of Helsinki as well as applicable institutional and governmental ethical research guidelines. Participants were informed about the study’s purpose, the risks and benefits of participating in the study, and they had the right to withdraw at any time while they filled out the questionnaire. Completion of the questionnaire indicated the agreement of participants to be part of the study. No identifiers were collected, and the data were reported in aggregated form. Access to the data remains restricted to the primary investigator.

### Data analysis

The data were managed in the Excel sheet and then exported to the Statistical Package for the Social Sciences (SPSS v30) which was used to analyze the data. The demographic characteristics of participants and item analysis were done using the descriptive statistics. Pearson’s correlation was used to assess the relationship between GSE, satisfaction with e-learning, and perceived academic performance. The next step was to include the significant variables in the linear regression model. The assumptions of the multiple linear regression (i.e., linearity, homoscedasticity, absence of autocorrelation and multicollinearity) were tested. The inspection of residual plots indicated that the linearity and homoscedasticity were met. The Durbin Watson results were between 1.5 and 2.5, indicating the absence of autocorrelation. The Variance Inflation Factor (VIF) was within the normal range (< 5) indicating the absence of multicollinearity. The Q–Q plot suggested that residuals were approximately normally distributed. The independent sample t-test was used to assess the mean differences in participants’ satisfaction with e-learning, GSE, and perceived academic performance according to demographics’ characteristics. The homogeneity of participants was assessed through Levene’s test (*p* > .05).

## Results

A total of 163 nursing students responded to the questionnaire (Table 1). The response rate could not be calculated because the survey link was distributed online through social media platforms, and the number of students who viewed or received the invitation could not be determined. The average age of participants was 26.8 (SD ± 6.7), and the majority of them were female (55.2%). Most participants are currently enrolling in the bachelor’s degree in nursing (*n* = 86), while graduate students represent approximately 45% of the total sample. The average GPA was 3.6 (SD ± 0.58). More than two thirds of participants have had the chance to attend training on e-learning.

**Table 1:** Demographic Characteristics of Participants (*n* = 163)

Variable (Range)	*n* (%) or M (SD)
Age	26.84 (6.79)
Gender	
Male	73 (44.8%)
Female	90 (55.2%)
Level of Education	
BSN	86 (52.8%)
MSN	48 (29.4%)
Ph.D.	25 (15.3)
Cumulative GPA	3.67 (0.58)
Attending Training Courses on E-learning	
Yes	135 (82.8%)
No	26 (16.0%)

### Satisfaction with e-learning, general self-efficacy, and perceived academic performance

Among the satisfaction dimensions (Table 2), the highest mean score was observed for satisfaction with assessment (M = 2.96, SD = 0.81), followed by satisfaction with generic skills and learning experiences (M = 2.94, SD = 0.82) and the lowest score was given to satisfaction with teaching (M = 2.86, SD = 0.90). The overall e-learning satisfaction mean score was 2.92 (SD = 0.63).

**Table 2:** Descriptive statistics of satisfaction with e-learning (teaching, assessment, and learning) (*n* = 163)

Subscale	Item Analysis	M	SD
Teaching	Overall satisfaction with teaching	2.86	.90
	1. My instructors had a thorough knowledge of the subject content.	2.79	1.09
	2. My instructors provided opportunities to ask questions.	2.91	1.44
	3. My instructors treated me with respect.	3.02	1.48
	4. My instructors understood my learning needs.	2.79	1.51
	5. My instructors communicated the subject content effectively.	2.74	1.45
	6. My instructors made the subject as interesting as possible.	2.93	1.40
Assessment	Overall satisfaction with assessment	2.96	.81
	7. I knew how I was going to be assessed.	3.07	1.11
	8. The way I was assessed was a fair test of my skills.	3.33	1.39
	9. I was assessed at appropriate intervals.	2.90	1.57
	10. I received useful feedback on my assessment.	2.63	1.38
	11. The assessment was a good test of what I was taught.	2.88	1.30
Generic Skills and learning experiences	Overall satisfaction with generic skills and learning experiences	2.94	.82
	12. My training developed my problem-solving skills.	3.46	1.13
	13. My training helped me develop my ability to work as a team member	3.39	1.50
	14. My training improved my skills in written communication.	3.09	1.60
	15. My training helped me to develop the ability to plan my own work.	2.60	1.61
	16. As a result of my training, I feel more confident about tackling unfamiliar problems.	2.45	1.48
	17. My training has made me more confident about my ability to learn.	2.63	1.60
	18. As a result of my training, I am more positive about achieving my goals]	2.93	1.54
	19. My training has helped me think about new opportunities in life.	3.01	1.56
Satisfaction with training	20. How would you rate, on average, your satisfaction with the overall quality of the training? [Overall, I was satisfied with the quality of this training.	3.82	1.17
Total satisfaction with e-learning		2.92	.63

Regarding GSE, participants generally reported moderate to high levels of general self-efficacy (Table 3). The item with the highest mean score was “I can usually handle whatever comes my way” (M = 2.93, SD = 0.71), followed closely by “If I am in trouble, I can usually think of a solution” (M = 2.92, SD = 0.80). The lowest mean score was for “I am confident that I could deal efficiently with unexpected events” (M = 2.36, SD = 0.94).

**Table 3:** Descriptive statistics for general self-efficacy (*n* = 163)

GSE Item Analysis	M	SD
1. I can always manage to solve difficult problems if I try hard enough.	2.47	0.90
2. If someone opposes me, I can find the means and ways to get what I want.	2.55	1.07
3. It is easy for me to stick to my aims and accomplish my goals.	2.53	0.91
4. I am confident that I could deal efficiently with unexpected events.	2.36	0.94
5. Thanks to my resourcefulness, I know how to handle unforeseen situations.	2.49	0.83
6. I can solve most problems if I invest the necessary effort.	2.80	0.86
7. I can remain calm when facing difficulties because I can rely on my coping abilities.	2.86	0.85
8. When I am confronted with a problem, I can usually find several solutions.	2.87	0.90
9. If I am in trouble, I can usually think of a solution.	2.92	0.80
10. I can usually handle whatever comes my way.	2.93	0.71

Academic performance items showed generally high mean scores, suggesting that participants perceive themselves as meeting or exceeding academic expectations (Table 4). The highest mean score was for “My performance is beyond demands” (M = 3.47, SD = 1.00), followed by “I adequately complete assigned duties” (M = 3.45, SD = 1.17). The lowest mean score was for “I meet the official performance requirements expected out of a student” (M = 3.13, SD = 1.00), which, while still positive, suggests a slightly lower agreement compared to other items.

**Table 4:** Descriptive statistics for perceived academic performance items (*n* = 163)

Item Analysis	M	SD
I meet the official performance requirements expected out of a student.	3.13	1.00
I adequately complete assigned duties.	3.45	1.17
I fulfill responsibilities specified (e.g., study, homework, readings, papers) in the course outline.	3.40	1.09
I perform tasks that are expected of me.	3.33	0.98
My performance is beyond demands.	3.47	1.00

### Mean differences in participants’ satisfaction with e-learning, general self-efficacy, and perceived academic performance according to demographics’ characteristics

The independent sample t-test was conducted to determine the mean differences in participants’ satisfaction with e-learning, general self-efficacy, and perceived academic performance (Table 5). The results indicated that female nursing students had greater satisfaction with e-learning (M = 3.04, *p*<.01), greater self-efficacy (M = 27.3, SD = 4.69, *p* < .05), and higher perceived academic performance (M = 3.47, SD = 0.69) comparing to male nursing students. Also, nursing undergraduate students had greater satisfaction with e-learning (M = 3.06, SD = 0.69, *p*<.01), and higher perceived academic performance (M = 3.46, SD= 0.67, *p* < .05) compared to graduate students. However, there was no significant difference in satisfaction with e-learning, general self-efficacy, and perceived academic performance for those who did and who did not attend training courses regarding e-learning.

**Table 5:** Mean differences in participants’ satisfaction with e-learning, general self-efficacy, and perceived academic performance according to demographics’ characteristics (*n* = 163)

Demographic Factors		Satisfaction with E-learning			General Self-Efficacy			Academic Performance		
		M (SD)	*t or f*	*p*	M (SD)	*t or f*	*p*	M (SD)	*t or f*	*p*
Gender	Male	2.77 (0.38)	*2.96*	0.002**	26.09 (2.9)	*2.04*	0.021*	3.21 (0.53)	*2*,*73*	0.004**
	Female	3.04 (0.75)			27.32 (4.69)			3.47 (0.69)		
Level of education	Undergraduate	3.06 (0.69)	3.0	0.002**	27.2 (4.50)	1.24	0.10	3.46 (0.67)	2.09	0.019*
	Graduate	2.77 (2.77)			26.4 (3.40)			3.25 (0.56)		
Attendance at training courses	Yes	2.93 (0.66)	0.65	0.25	27.0 (4.0)	1.49	0.06	3.37 (0.65)	0.79	0.215
	No	2.87 (0.44)			25.76 (3.66)			3.26 (0.54)		

Note: *p < .05; **p < .01

### The relationship between GSE, satisfaction with e-learning, and perceived academic performance

The Pearson’s product moment correlation was conducted to determine the correlation between general self-efficacy, satisfaction with e-learning, and academic performance (Table 6). General self-efficacy was significantly associated with satisfaction with e-learning (*r* = .579, *p* < .001) and academic performance (*r* = .511, *p* < .001), while satisfaction with e-learning was also significantly correlated with academic performance (*r* = .539, *p* < .001).

**Table 6:** Correlation between GSE, Satisfaction with e-learning, and Academic Performance

Variables	1	2	3
1. General Self-Efficacy	-		
2. Satisfaction with e-learning	0.579**	-	
3. Academic Performance	0.511**	0.539**	-

** *p*<.001

### The influence of general self-efficacy and satisfaction with e-learning on academic performance

Multiple linear regression was conducted to examine the associations of general self-efficacy and satisfaction with e-learning with perceived academic performance (Table 7). No missing data were identified for the variables entered into the analysis, as participants were required to respond to all items. Therefore, all 163 cases were included in the regression model.

**Table 7:** The influence of general self-efficacy and satisfaction with e-learning on the perceived academic performance (*n* = 163)

Model	Unstandardized Coefficients		Standardized Coefficients	t	*p*-value	95%CI	
	β	Std. Error	Beta			LL	UL
(Constant)	1.006	0.276		3.647	< 0.001	0.461	1.552
General Self-Efficacy	0.047	0.012	0.299	3.826	< 0.001	0.023	0.072
Satisfaction with E-learning	0.370	0.079	0.366	4.678	< 0.001	0.214	0.526
Model Summary	*F* (2,160) = 43.06, R^*2*^ = 0.35, adjusted R^*2*^ = 0.342, *p* = < 0.001						

*p< .001

The overall model was statistically significant, *F*(2, 160) = 43.06, *p* < .001, explaining 35.0% of the variance in perceived academic performance (*R*² = 0.350; adjusted *R*² = 0.342). Both general self-efficacy (*B* = 0.047, SE = 0.012, β = 0.299, *t* = 3.826, *p* < .001, 95% CI [0.023, 0.072]) and satisfaction with e-learning (*B* = 0.370, SE = 0.079, β = 0.366, *t* = 4.678, *p* < .001, 95% CI [0.214, 0.526]) were significantly and positively associated with perceived academic performance.

## Discussion

This study aimed to assess the relationships among self-efficacy, satisfaction with e-learning, and perceived academic performance in nursing students participating in virtual learning, guided by Albert Bandura’s theory of self-efficacy. Assessing both self-efficacy and e-learning satisfaction is crucial because these factors may significantly influence students’ academic performance.

Regarding e-learning satisfaction, findings demonstrated that nursing students reported relatively low satisfaction with e-learning. This finding aligns with previous research [5, 30], which found nursing students somewhat satisfied with e-learning before and during the COVID-19 pandemic. Conversely, a number of researchers conducted a qualitative study and found that nursing students were dissatisfied with e-learning. However, their sample comprised only twelve nursing students who were recruited from one nursing school [28].

Higher self-efficacy supports success in e-learning by positively impacting academic performance through enhancing motivation, self-regulation, and engagement [29]. Assessing its effects on e-learning satisfaction and academic performance is therefore critical. The present findings demonstrated moderate to high GSE levels, specifically participants nursing students generally perceive themselves as capable of coping with challenges and finding solutions. However, participants had lower confidence in their ability to deal efficiently with unexpected events. Our findings are consistent with a previous study [39] that reported elevated self-efficacy toward e-learning.

On the other hand, a study conducted in India among a sample of 332 nursing students found low self-efficacy toward e-learning [40], possibly because nursing education possesses profession-specific demands that are not limited to the acquisition of cognitive knowledge but involve the psychomotor skills, clinical reasoning, relational competence, and patient communication [41]. This may help explain why self-efficacy in nursing education does not develop uniformly across online learning contexts. In the present study, the association between self-efficacy and perceived academic performance suggests that students’ confidence in online learning may depend not only on their ability to manage theoretical content, but also on the extent to which the learning environment supports the broader practice-based demands of nursing education. This is particularly relevant in nursing education, where stronger self-efficacy may reflect greater readiness to engage with clinical learning demands and may be reinforced through mastery experiences provided by simulation and supervised practice.

Although e-learning is flexible and supports the acquisition of theoretical knowledge, these core competencies may not be entirely developed by online modalities only. Previous researchers reported that online education in health professions must not substitute the hands-on clinical education, but supplement it, especially on competencies related to skills, and patient-facing competencies [42, 43]. Similarly, nursing education literature emphasizes that developing clinical competence through practice-based learning and achieving patient-centered care competencies are central aims of nursing education [44].

The application of blended learning models incorporating e-learning with simulation and supervised clinical experiences to promote psychomotor skills acquisition and clinical reasoning is also supported by evidence of the current body of nursing education literature [45]. This may explain why more supportive and pedagogically appropriate learning environments are important for strengthening students’ academic confidence and perceived performance in nursing education. The blended and simulation-enhanced forms of learning therefore provide a more suitable and pedagogically reasonable model of nursing education than the purely online format.

Self-efficacy and e-learning satisfaction are paramount factors influencing academic performance among nursing students [17]. The present findings revealed significant correlations among GSE, e-learning satisfaction, and perceived academic performance. These results align with El-Gazar et al. [17], who reported a significant positive correlation between self-efficacy and academic performance. Similarly, a study that surveyed 10,092 graduate students across ten countries revealed that e-learning quality positively impacted academic performance, mediated by satisfaction [46].

Our study also identified a significant relationship between participants’ gender and e-learning satisfaction. It shows that female nursing students had greater satisfaction with e-learning. This aligns with a previous study conducted in Spain with 1185 students enrolled in online courses. That study reported greater satisfaction among female students than male students [47]. Additionally, a global systematic review further concluded that females in countries such as the United Kingdom, Spain, Austria, India, Chile, and mixed settings reported greater e-learning satisfaction than males [48]. Furthermore, additional research supported those findings [49], which align with the current findings. Notably, in the aforementioned studies, including our study, the female participants comprised two-thirds or more of the samples, potentially influencing outcomes.

Results further indicated significant gender differences in GSE, as female nursing students had greater satisfaction with GSE than male students. This aligns with a U.S. study showing higher e-learning self-efficacy among female students than males [48]. The current findings are consistent with studies reporting significantly higher self-efficacy among female students [48, 50]. Researchers also reported that there are generally no significant gender differences in e-learning outcomes except in a few countries. Females significantly outperformed males in Spain and the UK. In Austria, India, and mixed countries (Chile and Spain), females hold significantly more positive attitudes toward e-learning than males. In the USA, females present significantly higher self-efficacy than males [48].

Additionally, our findings demonstrated that undergraduate nursing students reported higher e-learning satisfaction than graduate students. To the best of our knowledge, no prior studies have determined an association between educational level and e-learning satisfaction, limiting comparisons. Finally, GSE and e-learning satisfaction were significantly associated with perceived academic performance. However, no studies have specifically examined these variables in relation to academic performance, limiting further comparisons.

### Limitations

Despite its novelty in exploring the influence of e-learning satisfaction and self-efficacy on perceived academic performance, this study faces several limitations. First, the use of a cross-sectional design and convenience sampling limits causal inference. Future studies are recommended to use longitudinal designs to better examine the relationships among e-learning satisfaction, self-efficacy, and academic performance. In addition, recruitment through social media may have introduced self-selection bias, as students who were more interested in e-learning or more engaged with online platforms may have been more likely to participate. This may have resulted in an overrepresentation of students who were more familiar and comfortable with digital learning environments, thereby potentially inflating the reported levels of e-learning satisfaction, self-efficacy, and perceived academic performance.

Second, the use of self-report measures may influence social desirability. Third, although the regression model revealed that 35% of the variation of the perceived academic performance was explained, this shows that a significant percentage of the variance is not explained. This could be due to the omission of the possible influential variables like digital literacy, socioeconomic background, previous exposure to e-learning, and teacher support. It is recommended that future studies use more holistic frameworks which incorporate the individual and contextual and instructional factors in a more holistic way to achieve greater success in explaining academic performance in the context of e-learning. Moreover, future studies could focus on the instructor-reported assessments to enhance the generalizability of the findings.

Finally, because the data were collected from institutions across the country, variation in e-learning modalities, technological infrastructure, and instructional quality is expected. As these institutional characteristics were not measured directly, their potential influence on the study variables could not be fully examined. Future research should compare specific e-learning modalities, such as live instructor-led and virtual classroom formats, and account for institutional factors to provide a more precise understanding of how e-learning satisfaction influences academic performance.

### Implications and future research directions

This study highlights the importance of identifying factors associated with e-learning satisfaction, GSE, and academic performance. Our findings demonstrated positive associations between selected sociodemographic factors, including gender and educational level, and GSE, e-learning satisfaction, and perceived academic performance.

Moreover, this study has several implications for nursing education. The positive association of self-efficacy and e-learning satisfaction with perceived academic performance suggests that nursing students may benefit from learning environments that strengthen confidence, engagement, and readiness for practice. Higher self-efficacy and greater satisfaction with the e-learning environment could help students to prepare better to clinical placements, as satisfaction with blended and e-learning methods has been associated with greater work preparedness due to improved confidence and self-efficacy [51]. Considering the acquisition of skills and safety, it has been reported in the literature that the use of e-learning and simulation modalities assists in the development of self-efficacy of clinical competencies which can be converted to safer practice when combined with the traditional training [52].

Further, the professional identity formation is also closely linked to self-efficacy, and the stronger the professional identity, the greater the levels of engagement and confidence in the role of professional learning [53]. This indicates that the application of educational strategies capable of developing self-efficacy can also be used to enhance the process of developing the professional identity in students. Lastly practice based training can be supplemented by synchronous and blended e-learning which are interactive, flexible learning opportunities that promote the reinforcement of theoretical knowledge and promote mastery experiences, but in-person clinical experiences are also necessary to realize complete development of hand on skills and contextual judgment.

Given that the sample reflects the typical demographic profile of nursing students (55.2% female and comprising both undergraduate and postgraduate participants), any observed demographic group differences should be interpreted with caution and not overgeneralized beyond the current sample. Despite these findings, future studies should compare undergraduate and graduate nursing students’ e-learning satisfaction and academic performance. Additionally, we recommend studies examining differences between e-learning and traditional in-person teaching methods. Qualitative studies should explore the challenges students encounter in e-learning environments. These findings can guide nursing educators in developing effective teaching strategies (e.g., incorporating self-efficacy-building activities or improving e-learning platforms) to promote student engagement and motivation in e-learning, thereby enhancing academic performance.

## Conclusion

In an e-learning context, learners’ confidence in their skills is reinforced and positive mastery experiences are provided, which can further boost self-efficacy and improve academic performance. The present results underscore the critical roles of self-efficacy and e-learning satisfaction in relation to academic performance, which is consistent with Bandura’s self-efficacy theory. Findings demonstrated significant relationship between GSE, e-learning satisfaction, and perceived academic performance. Moreover, female and undergraduate nursing students reported higher GSE and e-learning satisfaction than their counterparts. However, no differences emerged between students who attended e-learning training courses and those who did not regarding GSE, e-learning satisfaction, or academic performance. These insights can inform targeted interventions to strengthen these factors, ultimately improving nursing students’ academic performance and overall e-learning satisfaction.

## Data Availability

The datasets are available from the corresponding author [AI] upon reasonable request.
